# Unraveling molecular characteristics and functional exploration of panoptosis for prognosis stratification in lung adenocarcinoma: a tumor marker prognostic study

**DOI:** 10.1097/JS9.0000000000002968

**Published:** 2025-07-08

**Authors:** Kaibo Hu, Zhangyi Tu, Tianfeng Luo, Hongyi Lai, Guangyu Han, Leyang Xiao, Jitao Ling, Yixuan Chen, Deju Zhang, Wuming Wang, Jing Zhang, Peng Yu

**Affiliations:** a Department of Endocrinology and Metabolism, The Second Affiliated Hospital of Nanchang University, Nanchang, People’s Republic of China; b The Second Clinical Medical College, Nanchang University, Nanchang, People’s Republic of China; c Department of Cardiology, Fuwai Hospital, National Center for Cardiovascular Diseases, Chinese Academy of Medical Sciences and Peking Union Medical College, Beijing, People’s Republic of China; d The First Clinical Medical College, Nanchang University, Nanchang, People’s Republic of China; e Department of Anesthesiology, The Second Affiliated Hospital of Nanchang University, Nanchang, People’s Republic of China; f Food and Nutritional Sciences, School of Biological Sciences, The University of Hong Kong, Hong Kong; g Department of Thoracic Surgery, Jiangxi Provincial Chest Hospital, Nanchang, People’s Republic of China

**Keywords:** lung adenocarcinoma, machine learning, PANoptosis, vasopressin, YWHAG

## Abstract

**Background:**

As a unique cell death modality mediated by multifaceted PANoptosome complexes, PANoptosis plays a crucial role in the development, invasion, and drug resistance of cancers. However, there is a lack of mechanisms for the PANoptosis in lung adenocarcinoma (LUAD).

**Methods:**

More than 1500 biopsy samples of LUAD and other cancers were collected from diversified cohorts in various databases. Ten machine-learning methodologies were combined into 101 algorithm combinations to establish the prognostic model. The landscape of tumor immune microenvironment (TIME) and response to immunotherapy were assessed across the bulk-transcriptome profile utilizing different algorithms. Single-cell RNA sequencing unveiled one crucial gene as the risk factor of LUAD, which was verified and explored the potential biological mechanisms *in vitro* and *in vivo*.

**Results:**

In our research, the PANoptosis Score (PAN Score) can be regarded as an independent prognostic factor for LUAD, which outperformed other clinical features and previously published literatures. Integrating PAN Score with other clinical features into a nomogram enhanced the predictive accuracy. Meanwhile, patients with high PAN Score exhibited a suppressed TIME, less tumor-infiltrated lymphocytes, and resistance to immunotherapy and chemotherapy. As the crucial contributor of PAN Score, deficiency of 14-3-3γ (YWHAG) impaired LUAD progression significantly *in vitro* and *in vivo*, enhanced the sensitivity to chemotherapy, as well as activated the PANoptosis via decreasing the concentration of vasopressin.

**Conclusions:**

Briefly, PAN Score is a robust biomarker for the prediction of prognosis and therapy response in LUAD patients. The YWHAG-vasopressin-PANoptosis axis may become the potential therapeutic target for LUAD.

## Background

Lung cancer is one of the most common cancers worldwide and a leading cause of cancer-related death^[1]^. It is generally divided into two main types: small cell lung cancer (SCLC) and non-small cell lung cancer (NSCLC)^[2]^. NSCLC is more common, accounting for 80%–85% of all lung cancer cases. It is further divided into three main subtypes: lung adenocarcinoma (LUAD), lung squamous cell carcinoma, and large cell carcinoma, each of which has a different treatment approach and prognosis^[3]^. Early stage of LUAD often has no obvious symptoms, which makes early detection difficult. However, as the disease progresses, patients may experience symptoms such as persistent coughing, coughing up blood, chest pain, difficulty breathing, weight loss, and persistent fatigue^[4]^. And the survival rate of advanced LUAD patients was extremely low (only 16%–20%) because of the poor efficiency of the extent therapeutic strategies^[5]^. Therefore, there is an urgent need to elucidate the molecular mechanisms of the development of LUAD, identify effective prognostic biomarkers, and develop combination strategies to enhance chemosensitivity, thereby minimizing resistance, improving the prognosis, and extending the survival of patients with LUAD.

PANoptosis is a broad pattern of apoptosis involving multiple cell death pathways and mechanisms, resulting in programmed cell death^[6]^. This mode of apoptosis includes not only classical apoptosis, but also other forms of programmed cell death, such as pyroptosis and necroptosis^[7]^. PANoptosis is characterized by the synergistic action of multiple apoptotic signaling pathways, including endogenous apoptotic pathways (such as mitochondrial pathways) and exogenous apoptotic pathways (such as death receptor pathways), which jointly regulate the process of cell death^[8]^. Abnormal regulation of PANoptosis in malignant tumors may lead to overgrowth of tumor cells, drug resistance, and treatment failure^[9]^. An increasing amount of evidence suggests that characterizing PANoptosis patterns in cancers can predict survival rates and response to immunotherapy and chemotherapy, emphasizing the importance of understanding PANoptosis and its mechanisms for identifying new therapeutic targets and strategies to improve cancer treatment efficacy and patient prognosis^[10–12]^. However, the effects and underlying mechanisms of PANoptosis in LUAD remain largely unexplored.

Although PANoptosis has been increasingly recognized as a critical regulator of cancer progression and treatment resistance, its molecular characteristics and functional implications in LUAD remain poorly understood. Prior studies have largely focused on isolated cell death pathways or limited gene sets, often neglecting the integrative nature of PANoptosis. Moreover, existing prognostic models for LUAD lack the rigor of multi-algorithm machine-learning frameworks and fail to account for the dynamic interplay between PANoptosis and the tumor immune microenvironment (TIME). Furthermore, our study systematically characterized PANoptosis-related genes (PRGs) in LUAD using multi-omics data and advanced machine-learning methodologies and developed a robust prognostic model validated across six independent cohorts, outperforming previously published prognostic signatures. In addition, we identified the 14-3-3γ (YWHAG)-vasopressin (VP)-PANoptosis axis as a novel therapeutic target, with experimental validation demonstrating its role in enhancing chemosensitivity and inhibiting LUAD progression. By elucidating these mechanisms, our work not only advances the understanding of PANoptosis in LUAD but also provides an actionable biomarker for prognosis stratification and personalized therapy. Meanwhile, our methodology aligns with the TITAN guidelines^[13]^ to ensure rigorous and transparent reporting of AI components.

## Methods

This work has been reported in line with the REMARK criteria^[14]^.

### Data source and download

PRGs (*n* = 66) were curated from MSigDB, GeneCards, KEGG, and literature. Six independent LUAD cohorts (*n* = 1594) were obtained from TCGA and GEO databases. The inclusion criteria and details of cohorts are presented in Supplemental Digital Content Methods, available at: http://links.lww.com/JS9/E599, and Supplemental Digital Content Table S1, available at: http://links.lww.com/JS9/E601, Supplemental Digital Content Table S2, available at: http://links.lww.com/JS9/E602.

**HIGHLIGHTS**

- The study offers a groundbreaking approach to predict lung adenocarcinoma (LUAD) progression, response to therapy, and patient survival rates with heightened accuracy derived from PANoptosis.
- This research establishes the PAN Score as an innovative and robust prognostic biomarker via serious procedure and comparison with other models.
- The tight relationship between PANoptosis and tumor immune microenvironment is confirmed by multi-omics investigation.
- We first indicated that 14-3-3γ (YWHAG) deficiency impairs LUAD progression, sensitizes cells to chemotherapy, and triggers PANoptosis, underscoring its potential as a therapeutic target.
- YWHAG-vasopressin-PANoptosis axis was first ensured to illustrate the role of PANoptosis in LUAD.

### Establishment and validation of the prognostic signature

Univariate Cox regression identified prognostic PRGs. A 101-algorithm framework integrating 10 machine learning methods (e.g. CoxBoost, Lasso, RSF, SVM) was applied to TCGA-LUAD and five validation cohorts. The optimal algorithm was selected by maximizing the C-index. The final PANoptosis Score (PAN Score) was calculated as: PANoptosis Score (PAN Score) = Σ (Ci * Ei). The *Ci* indicates the coefficients of each PRG and the *Ei* represents the expression value of each PRG. Patients were dichotomized (high/low risk) using the “maxstat”-determined cutoff. Model performance was assessed via Kaplan–Meier analysis, time-dependent receiver operating characteristic (ROC) curves, and *C*-index comparison against published models. The details of model construction and validation can be seen in Supplemental Digital Content Methods, available at: http://links.lww.com/JS9/E599.

### Construction and evaluation of the nomogram combined with various clinical indexes

Multivariate Cox regression (integrating PAN Score, age, gender, T, N, M, and pathological stage) generated a prognostic nomogram, validated by calibration and clinical decision curves (see Supplemental Digital Content Methods, available at: http://links.lww.com/JS9/E599).

### Description of the tumor microenvironment

Immune infiltration was quantified using CIBERSORT, ESTIMATE, quanTIseq, TIP, xCell, MCP-counter, TIMER, and ssGSEA. Tumor-infiltrated lymphocytes (TILs) were mapped via TCGA TIL Maps. The gene sets used in this section and comprehensive procedures are exhibited in Supplemental Digital Content Methods, available at: http://links.lww.com/JS9/E599 and Supplemental Digital Content Table S3, available at: http://links.lww.com/JS9/E603, Supplemental Digital Content Table S4, available at: http://links.lww.com/JS9/E604.

### Investigation of the novel therapy strategies

Differential expression of immune checkpoints (ICKs) (e.g. PD-1, CTLA-4) and TIDE scores (TIDE <0: responder) were compared between PAN Score groups. External validation used TIGER datasets. GDSC data analyzed via “oncoPredict” for chemotherapy response. Details can be seen in Supplemental Digital Content Methods, available at: http://links.lww.com/JS9/E599.

### Single-cell analysis

A single cell dataset, GSE131907, processed using Seurat 4.0. Cell clusters were annotated manually. The comprehensive process of single cell analysis is exhibited in Supplemental Digital Content Methods, available at: http://links.lww.com/JS9/E599.

### Pan-cancer analysis

PAN Score prognostic utility was tested across 33 other cancers in the TCGA (*n* = 10 110). The details of analysis and 33 cancer cohorts are provided in the Supplemental Digital Content Methods, available at: http://links.lww.com/JS9/E599, and Supplemental Digital Content Table S5, available at: http://links.lww.com/JS9/E605.

### Experiments in vitro and vivo

We conducted a series of experiments in the human, cell, and animal level. The details, including primers, antibodies, etc, of these experiments can be seen in Supplemental Digital Content Methods, available at: http://links.lww.com/JS9/E599, and Supplemental Digital Content Table S6, available at: http://links.lww.com/JS9/E606, Supplemental Digital Content Table S7, available at: http://links.lww.com/JS9/E607.

### Statistical analysis

The statistical analysis was conducted using GraphPad Prism 9.5 Software (GraphPad Software, La Jolla, CA, USA). The level of statistical significance was set at a *P <* 0.05. *, **, ***, and **** represent the *P* < 0.05*, P* < 0.01*, P* < 0.001, and *P* < 0.0001, respectively. The unpaired *t*-test was for two sets comparison and analysis of variance test was for more than two sets comparison.

## Results

### Construction and validation of the PAN score prognostic model

**Figure 1.**
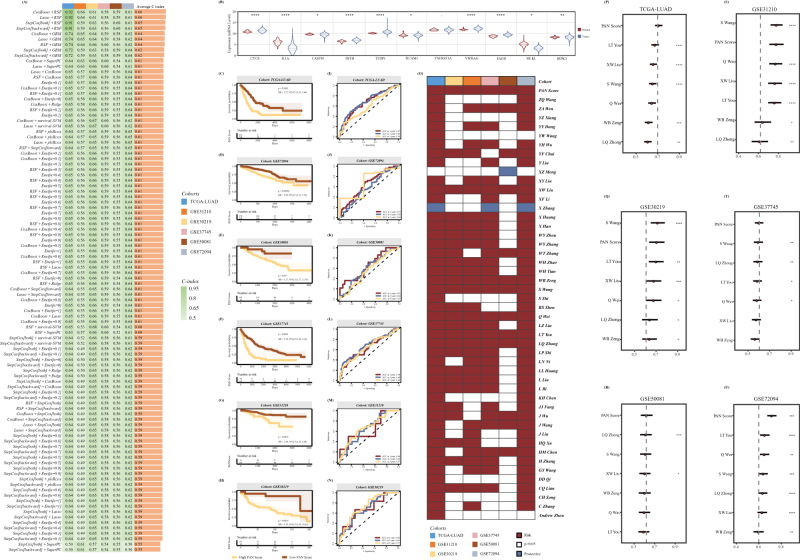
Exploitation and verification of the prognostic model: PANoptosis Score (PAN Score). (A) C-indices of 101 machine learning algorithm combinations across all the six cohorts. (B) Expression level of the 11 elements in the PAN Score between tumor and normal groups in the TCGA-LUAD cohort. (C–H) KM curves of the PAN Score across multiple cohorts. (I–N) Time-dependent ROC curves of the PAN Score across multiple cohorts in the 1-, 3-, and 5-year. (O) Prognostic heat map indicates the results of the univariate-Cox regression analysis for PAN Score and previously published models across multiple cohorts. (P–U) Comparison of the *C*-indices between PAN Score and other six excellent models. “-” indicates *P*-value >0.05, *P-value <0.05, **P-value <0.01, ***P-value <0.001, **** *P*-value <0.0001, by unpaired *t*-test.

According to the previously published literature, a total of 66 PRGs were extracted, of which 26 participates in *pyroptosis*, 32 are related to *apoptosis*, and 8 are associated with *necroptosis* (Supplemental Digital Content Figure S1A, available at: http://links.lww.com/JS9/E600). Then, combining the expression value of the above 66 PRGs in our training cohort TGCA-LUAD with the corresponding survival information, the univariate Cox regression was performed to further select several crucial prognostic PRGs. As a result, a total of 12 prognostic PRGs were identified, which were regarded as the input elements to generate our multiple prognostic models of LUAD, including BMF, CASP10, CYCS, DFFB, FADD, IL1A, MLKL, RIPK3, TICAM1, TFDP1, TNFRSF1A, and YWHAG (Supplemental Digital Content Figure S1B, available at: http://links.lww.com/JS9/E600). The comprehensive results of the univariate Cox regression are exhibited in Supplemental Digital Content Table S8, available at: http://links.lww.com/JS9/E608. The mutation status of the above 12 PRGs is exhibited in Supplemental Digital Content Figure S2, available at: http://links.lww.com/JS9/E600, in which TFDP1 possessed the highest mutation rate. Furthermore, via assessing in the training set and other five external validation cohorts, the prognostic model constructed through the integration of CoxBoost and RSF was selected as the most appropriate model with the highest average *C*-index (0.67) in all the 101 algorithm patterns, in which 11 prognostic PRGs were comprised except BMF (Fig. 1A). The details of each pattern’s *C*-index in the training and validation cohorts were listed in Supplemental Digital Content Table S9, available at: http://links.lww.com/JS9/E609. And Figure 1B revealed the expression level of these 11 PRGs between normal and tumor in the TCGA-LUAD cohort.

Utilizing this model, the PAN Score of each patient across the six various cohorts was calculated, whose calculation formula was as follows: *PAN Score* = Exp _(CYCS)_ *0.0373283 + Exp _(IL1A)_ *0.08280205 - Exp _(CASP10)_ *0.11229597 - Exp _(DFFB)_ *0.09689826 + Exp _(TFDP1)_ *0.12742004 + Exp _(TICAM1)_ *0.14649184 + Exp _(TNFRSF1A)_ *0.06954093 + Exp _(YWHAG)_ *0.14757923 + Exp _(FADD)_ *0.06317395 + Exp _(MLKL)_ *0.08073902 - Exp _(RIPK3)_ *0.13219889. And the KM curve in the TCGA-LUAD cohort confirmed the crucial prognostic characteristic of PAN Score (*P* < 0.05, Fig. 1C). Besides, the consistent phenomenon also occurred in all the validation cohorts, which further emphasizes the accurate prognostic role of the PAN Score (Fig. 1D–H). Furthermore, to better demonstrate the discrimination of the PAN Score, the time-dependent ROC curves were exhibited, with 1-, 3-, and 5-year AUCs of 0.67, 0.69, and 0.59 in the TCGA-LUAD cohort; 0.65, 0.58, and 0.69 in the GSE72094 cohort; 0.65, 0.60, and 0.56 in the GSE50081 cohort; 0.64, 0.68, and 0.64 in the GSE37745 cohort; 0.64, 0.66, and 0.67 in the GSE31210 cohort; and 0.68, 0.69, and 0.73 in the GSE30219 cohort, respectively (Fig. 1I–N).

To more comprehensively delineate the advantages of PAN Score model within other prognostic models in LUAD, we collected all 47 publicly published literature on constructing prognostic and predictive mRNA-models via machine learning previously and compared the clinical performance with the PAN Score (Supplemental Digital Content Table S10, available at: http://links.lww.com/JS9/E610). We performed KM prognostic analysis for PAN Score and all the previous models in all the six cohorts included in our research and indicated that only seven of the models play a consistent and significant prognostic role across all the six datasets (Fig. 1O). Subsequently, we calculated and compared the C-index of these seven models in our six training and validation cohorts. Intriguingly, the PAN Score stamped better property than the majority of published models in all six datasets, which further revealed the stability and superiority of the PAN Score (Fig. 1PU).

### Evaluation of the prognostic efficiency according to PAN score and clinical characteristics

**Figure 2.**
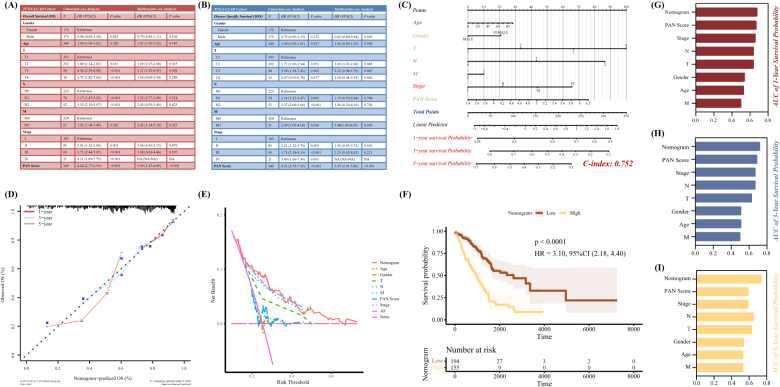
Prognostic prediction based on the PANoptosis Score (PAN Score) and other clinical features. Univariate- and multivariate-Cox regression analysis emphasized PAN Score as an independent prognostic factor according to (A) overall survival (OS) time and (B) disease-specific survival (DSS) time. (C) A nomogram derived from the combination of PAN Score and other clinical features in the TCGA-LUAD cohort. (D) Calibration curves in the 1-, 3-, and 5-year, (E) Clinical decision curve, and (F) KM curve of the established nomogram. (G-I) Comparison of the area under the curve (AUC) in the 1-, 3-, and 5-year survival probability among the nomogram and other elements.

Seeing the independent prognostic accuracy of PAN Score in these training and validation cohorts, the relationships between the recognized clinical factors, such as age, gender, T, N, M, and pathological stage and PAN Score were worthy to be elucidated to establish an enhanced comprehensive model for survival prediction. In the TCGA-LUAD cohort, the univariate and multivariate Cox regression analysis demonstrated that both OS and DSS information of LUAD patients exhibited a significant association with clinical parameters, like *PAN Score*, gender, and N (Fig. 2A and B). Then, to avoid the one-sidedness of using *PAN Score* as an independent prognostic indicator, we integrated various clinical information and *PAN Score* to perform multivariable-Cox and step-wise regression analysis to construct a comprehensive nomogram scoring model in the TCGA-LUAD cohort, whose calculated formula and corresponding coefficients were listed in Table 1 (*C*-index: 0.752; Fig 2C). And the calibration curve described the predictive reliability of the nomogram model in the 1-, 3-, and 5-year (Fig. 2D). Moreover, the clinical decision curves indicated the superior performance of nomogram score than these independent prognostic indexes included in the research (Fig. 2E). Meanwhile, the KM curve indicated the nomogram score as a risk prognostic factor in LUAD (*P <* 0.0001, Fig 2F). To further validate the predictive prognostic characteristic of the nomogram model, the time-dependent ROC curve was exhibited and the AUCs of 0.68 in 1-year, 0.72 in 3-year, and 0.74 in 5-year (Fig. 2G–I).

**Table 1:** The formula and corresponding coefficients to calculate the score of each LUAD patient in the nomogram

Features	Calculation formula and corresponding coefficients	
Age	0.314991389 * age	
Gender	Male	0
	Female	20.6958
T	1	0
	2	33.33333
	3	66.66667
	4	100
N	0	0
	1	43.23227
	2	86.46453
M	0	0
	1	10.08796
Stage	I	0
	II	21.89503
	III	43.79006
	IV	66.68509
PAN Score	27.142050118 * PANScore - 92.2829704	
1-year survival probability	−6e-09 * points ^3–1.623e-06 * points ^2 - 0.000323661 * points + 0.957616369	
3-year survival probability	2.6e-08 * points ^3–1.6095e-05 * points ^2 + 0.000167422 * points + 0.825109197	
5-year survival probability	3.5e-08 * points ^3–1.3238e-05 * points ^2 - 0.00157023 * points + 0.732251326	

### Validation and expansion of PAN score in the pan-cancer cohort

First, the relationship between our 12 PRGs and 9 cancer-associated pathways was calculated, including TP53, WNT, RTK-RES, TGF-β, PI3K, Nrf2, Notch, Hippo, and Cell cycle, which revealed the key role of our model in cancer development (Supplemental Digital Content Figure S3A, available at: http://links.lww.com/JS9/E600). Considering the precise predictive role of PAN Score in the prognosis of LUAD, we further excavate the characteristic of our model in predicting the prognosis of cancer patients in the Pan-cancer cohort, including 10 467 patients of 33 kinds of cancers. Specifically, the univariate-Cox regression analysis indicated that the PAN Score can be regarded as a risk factor in 10 cancers, including ACC, CESC, HNSC, KICH, LGG, LIHC, MESO, PRAD, THYM, and UVM (Supplemental Digital Content Figure S3B, available at: http://links.lww.com/JS9/E600). Moreover, the KM curves of PAN Score in each cancer were exhibited and PAN Score was considered as a risk factor in 15 kinds of cancers (Supplemental Digital Content Figure S3C–Q, available at: http://links.lww.com/JS9/E600). Overall, the above finding in large-population cohorts claimed the accurate predictive role of PAN Score across the prognosis of various cancer types.

### *Remolding of the TIME* mediated *by PAN score in LUAD*

**Figure 3.**
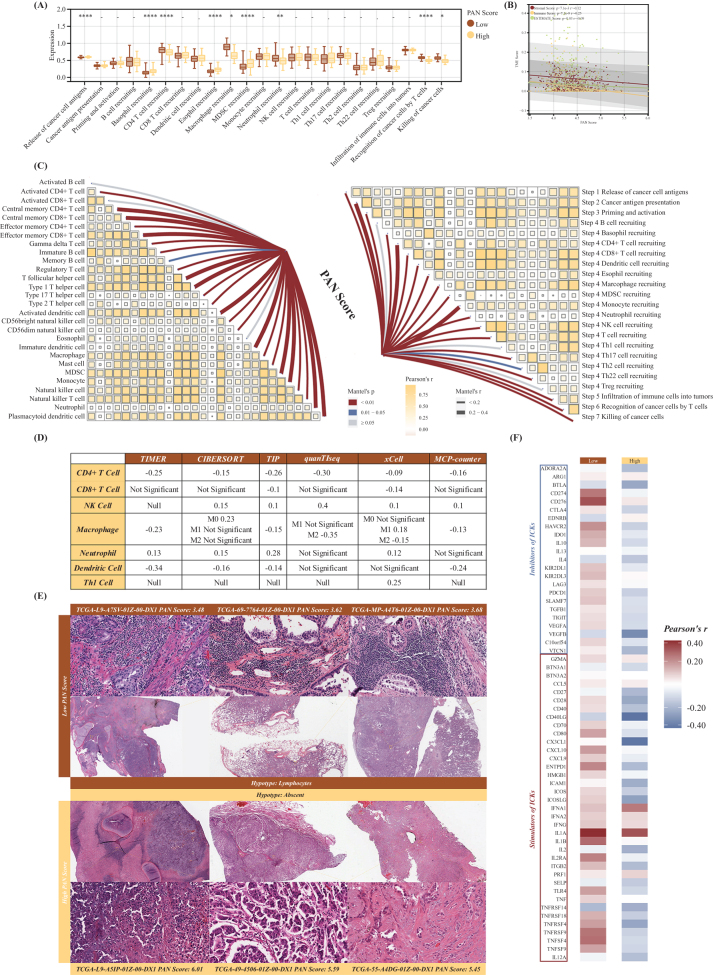
Remolding the tumor immune microenvironment within the regulation of PANoptosis Score (PAN Score). (A) Box plot reveals the difference of diversified steps of the tumor immunity cycle between patients with high- and low-PAN Score in TCGA-LUAD cohort. (B) Correlation between the PAN Score and the three indices in ESTIMATE algorithm, including Stromal Score, Immune Score, and ESTIMATE Score. (C) Correlation between the PAN Score and the infiltration score of 28 kinds of immune cells calculated via ssGSEA (left); Correlation between the PAN Score and the weights of steps the tumor immunity cycle (right). (D) Correlations between the PAN Score and the infiltration score of six tumor-infiltrated immune cells calculated by six independent algorithms. (E) Histopathological images from TCGA exhibit the HE staining differences between the high- and low-PAN Score group. (F) Correlations between the PAN Score and the expression of ICKs’ regulators in the low- and high-PAN Score group.

Increasingly, research has confirmed that the TIME in LUAD was characterized through diversified aspects, such as the existence of immune regulators, the dynamic infiltration of tumor immune cells, and the specific expression of ICKs. Moreover, the prominent association between PANoptosis and TIME has been indicated previously. Herein, we explored the potential characteristic of PAN Score in regulating these diversified immune aspects, thoroughly. Nowadays, the recognized regular Cancer Immunity Cycle reveals the dynamic participation of various immune modulators like chemokines, antigens, cytokines, and diversified immune cells. For the LUAD patients with lower PAN Score, the principal part of the process in the above cycle was significantly activated, including CD4 T cell recruiting (Step 4), Macrophage recruiting (Step 4), Neutrophil recruiting (Step 4), Recognition of cancer cells by T cells (Step 6), Killing of cancer cells (Step 7). However, only the Step 1, Release of cancer cell antigens, exhibited a higher level in the patients with higher PAN Score, which emphasized the malignant characteristic in the high PAN Score bunch (Fig. 3A). Subsequently, we found that there existed a significantly negative correlation between the PAN Score and the three indexes within the result of ESTIMATE algorithm, including stromal score, immune score, and ESTIMATE score (Fig. 3B). Meanwhile, Figure 3C revealed that both the score of various steps in the Cancer Immunity Cycle and the infiltration score of 28 different immune cells was significantly associated with the PAN Score in LUAD. And to investigate the role of PAN Score in the TIME of LUAD, six various algorithms were conducted, including TIMER, CIBERSORT, TIP, quanTIseq, xCell, and MCP-counter. Being consistent with the above result, the PAN Score was negatively correlated with the infiltration of immune cells, demonstrating that patients with lower PAN Score may be regarded as “immune-hot” LUAD while patients with higher PAN Score as “immune-cold” LUAD (Fig. 3D). In addition, the histopathological biopsies also confirmed the elevated infiltration of immune cells in patients with low PAN Score, further emphasizing the crucial negative role of PAN Score in the TIME of LUAD (Fig. 3E). Meanwhile, deep learning methods were utilized to outline the TILs in the histopathological biopsies of LUAD. As exhibited in Supplemental Digital Content Figure S4, available at: http://links.lww.com/JS9/E600, the percentage of TILs was significantly higher in patients with low PAN Score, which was consistent with the above analysis. And the correlation between the expression of ICKs’ regulators and PAN Score was developed in Figure 3G, comprehensively excavating the characteristic of PAN Score in shaping the TIME of LUAD.

### Efficiency in predicting the response of immunotherapy via PAN score

Via the thorough exploration of the TIME regulated by PAN Score in LUAD, we conjectured that LUAD patients with lower PAN Score and “immune-hot” tumor type are more likely to get advantages from the implement of immunotherapy. Given the above suppose, the TIDE analysis was performed to exhibit the various potential responses to immunotherapy in our training cohort, TCGA-LUAD. Specifically, the decreased TIDE score in the low PAN Score group support the beneficial result during the immunotherapy (Supplemental Digital Content Figure S5A, available at: http://links.lww.com/JS9/E600). Meanwhile, other indices as the result of TIDE analysis, including TMB, MSI, Dysfunction score, and Exclusion score, revealed the heterogeneity between patients with high and low PAN Score within receiving immunotherapy (Supplemental Digital Content Figure S5B–E, available at: http://links.lww.com/JS9/E600). Interestingly, when we defined whether the TIDE score was more than 0 as the outcome of the KM curve and plotted it, we found that the outcome between patients with high and low PAN Score exhibited significant difference (Supplemental Digital Content Figure S5F, available at: http://links.lww.com/JS9/E600). Similarly, the response rate in the low PAN Score group (40.10%) was higher than that in the high PAN Score group (23.21%) (Supplemental Digital Content Figure S5G, available at: http://links.lww.com/JS9/E600). Meanwhile, most ICKs’ expression exhibited a higher trend in the low PAN Score group with statistical significance (*P <* 0.05, Supplemental Digital Content Figure S5H, available at: http://links.lww.com/JS9/E600). All the above observations emphasized the crucial role of PAN Score in the prediction of immunotherapy for LUAD. In addition, three external immunotherapy cohorts of LUAD were selected to validate the predictive role of PAN Score in the immunotherapy of LUAD. Among all the three cohorts, the PAN Score was higher in the no-response group (NR) while the response rate was higher in the patients with response to immunotherapy (Supplemental Digital Content Figure S5I–N, available at: http://links.lww.com/JS9/E600). Moreover, to exhibit the ascendant characteristic of PAN Score in the prediction of immunotherapy, we included three immunotherapy cohorts of other cancer, including cutaneous melanoma, glioblastoma multiforme (GBM), and renal cell carcinoma, in which the similar trend of PAN Score between the NR and R was found, as well as the response rate between the high and low PAN Score group (Supplemental Digital Content Figure S5O–T, available at: http://links.lww.com/JS9/E600). Thus, comprehensively integrating the aforementioned findings, a potential hypothesis can be pointed out that the immunotherapy might generate more protective outcomes in the LUAD patients with lower PAN Score.

### In silico assessment for the chemotherapy efficiency within the PAN score

In order to ensure the candidate with high efficiency when receiving chemotherapy, we calculate the IC50 of 198 chemotherapeutics in the TCGA-LUAD cohort and compared the difference of IC50 between the high – and low-PAN Score group. According to the criteria *P*-value <0.05, a total of 19 drugs were identified, whose IC50 were all lower in patients with low PAN Score (Supplemental Digital Content Figure S6A–T, available at: http://links.lww.com/JS9/E600). The above findings indicated that patients with low PAN Score may benefit from the implement of chemotherapy.

### Identifying YWHAG as a risk factor of LUAD

**Figure 4.**
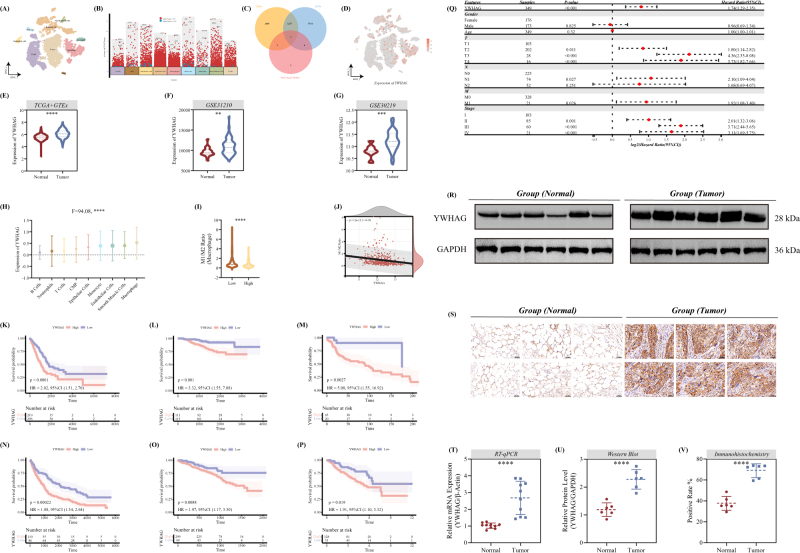
Identification and validation of the crucial prognostic factor for LUAD through scRNA-seq and in-house cohort. (A) A T-distributed Stochastic Neighbor Embedding (tSNE) plot of nine cell clusters. (B) A Manhattan plot pinpointed the significantly differentially expressed genes (DEGs) in each cell clusters. (C) Intersection among DEGs, LEGs, and the 11 elements in the PAN Score model exhibited in the Venn gram. (D) A tSNE plot of the normalized expression of YWHAG in each cell cluster. Boxplots indicated the expression level of YWHAG between tumor and normal group in the LUAD cohorts: (E) TCGA-LUAD and GTEx, (F) GS31210, and (G) GSE30219. (H) Expression difference of YWHAG in each cell cluster. (I) The difference of M1/M2 Ratio between the low- and high-PAN Score group. (J) The correlation between the expression of YWHAG and the M1/M2 Ratio. KM curves exhibited the superior prognostic role of YWHAG for LUAD across all the six cohorts: (K) TCGA-LUAD, (L) GSE31210, (M) GSE30219, (N) GSE37745, (O) GSE72094, and (P) GSE50081. (Q) Univariate-Cox regression analysis emphasized YWHAG as an independent prognostic factor of LUAD combined with other clinical features. Expression of YWHAG in the protein level between tumor and normal group in our in-house cohort detected via (R) western blot (WB, *n* = 6/group) and (S) immunohistochemical (IHC, *n* = 6/group) staining. Boxplots exhibited the statistical results of the mRNA and protein expression of YWHAG via (T) RT-qPCR (*n* = 10/group), (U) WB (*n* = 6/group), and (V) IHC (*n* = 6/group). **P-value <0.01, ****P-value <0.0001, by unpaired *t*-test.

To further pinpoint potential therapeutic targets specific to LUAD, we evaluate the level of PAN Score in various cell types. First, a scRNA-seq dataset, GSE131907, including 11 tumor samples, was selected in our analysis. Then, a total of nine cell clusters, including B cells, common myeloid progenitor cells, endothelial cells, epithelial cells, macrophages, monocytes, neutrophils, smooth muscle cells, and T cells (Fig. 4A). According to the criteria adjust *P*-value <0.01 and |log2FC| >1, the DEGs among each cell cluster were acquired and exhibited in Figure 4B and meanwhile, we explored the specific expressed genes related to LUAD (LEGs) between tumor and normal group in our training cohort. Via intersecting the DEGs, LEGs, and the 11 elements in our PAN Score Model, only YWHAG was identified as the most crucial factors (Fig. 4C) and the expression of YWHAG in each cell was also indicated in Figure 4D. And on the basis of canSAR database, YWHAG possessed 89% cancer score, which emerges as a potential target of patients with high PAN Score.

Moreover, we further explored the expression of YWHAG between tumor and normal groups in the TCGA-LUAD, GTEx, GSE31210, and GSE30219 cohorts, in which there was a significantly high expression in the tumor group (Fig. 4E*–*G). In the single-cell level, we compared the expression of YWHAG in each cell cluster and YWHAG exhibited the highest expression in macrophages (Fig. 4H). Strikingly, the M1-like/M2-like macrophages ratio (M1/M2 ratio) was significantly higher in the low-PAN Score group with significant negative correlation to the expression level of YWHAG, further emphasizing the cancerous role of PAN Score and YWHAG (Fig. 4I and J). Meanwhile, we collected LUAD and adjacent samples for the external validation of YWHAG. And for the prognostic role of YWHAG, we exhibited the KM curves of YWHAG in all of our training, testing, and validation cohorts, all of which demonstrated that patients with high-expressed YWHAG possess a poor prognosis (Fig. 4K*–*P). Combined with other clinical factors, YWHAG also exhibited crucial prognostic characteristic (Fig. 4Q). In addition, both in the mRNA and protein level, we indicated the high expression trend of YWHAG in the tumor group, which was consistent with the results of public datasets (Fig. 4R*–*V).

### Knockdown of YWHAG inhibits the activity of LUAD in vitro and in vivo

**Figure 5.**
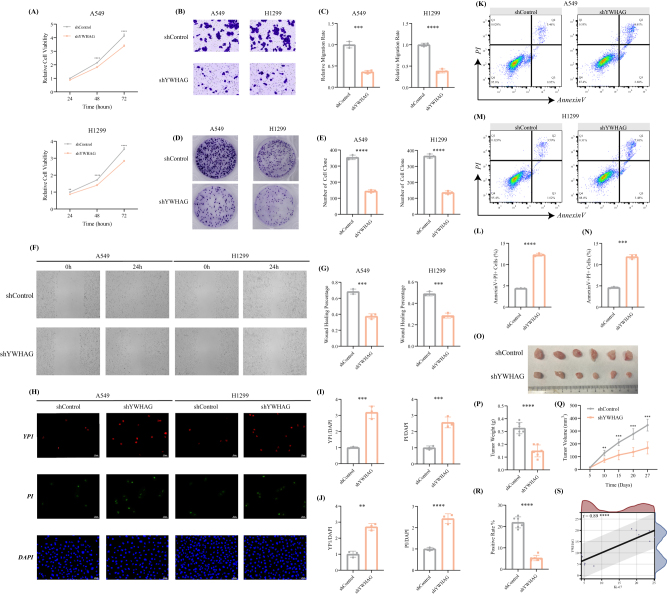
Characteristic of YWHAG knockdown in the inhibition of LUAD. (A) Cell viability assays indicated the cell proliferation phenotype of LUAD in the labelled cell groups. (B, C) Transwell assays exhibited the invasion phenotype of LUAD in the labelled cell groups (*n* = 3/group). (D, E) Clonoy formation assays of LUAD in the labelled cell groups (*n* = 3/group). (F) Representative images of wound healing exhibited the migration phenotype of LUAD in the labelled cell groups (*n* = 3/group). (G) Relative quantification of the width ratio of the wound in the labelled cell groups. (H) Representative images of immunofluorescence showed the YP1+ cells (red) and PI+ cells (green) in the labelled cell groups (*n* = 3/group). Relative quantification of the proportion of YP1+ cells and PI+ cells in (I) A549 and (J) H1299 cell lines. (K, L) Representative images for flow cytometric analysis of apoptosis in the labelled cell groups (*n* = 3/group). Relative quantification of the percentage of AnnexinV+PI+ cells in (M) A549 and (N) H1299 cell lines. (O) Visible size of the subcutaneous tumor models after YWHAG inhibition or control LUAD cell lines (*n* = 6/group). Relative quantification of the (P) tumor weight and (Q) tumor growth rate of the xenograft models. (R) The positive rate of Ki-67 of the subcutaneous tumor models after YWHAG inhibition or control LUAD cell lines. (S) Correlations between the positive of Ki-67 and YWHAG of the subcutaneous tumor models after YWHAG inhibition or control LUAD cell lines. **P-value <0.01, ***P-value <0.001, ****P-value <0.0001, by unpaired *t*-test or Tukey HSD test.

To unravel the role of YWHAG in LUAD, we first constructed three shRNAs and selected the canonical one to silence the expression of YWHAG in A549 and H1299 cells, in which the shYWHAG-1 was regarded as the optimal sequence according to the results of RT-qPCR and WB (Supplemental Digital Content Figure S7A–D, available at: http://links.lww.com/JS9/E600). And the knockdown of YWHAG inhibited the malignant phenotypes of the above two cell lines, including proliferation (Fig. 5A), invasion (Fig. 5B and C), colony formation (Fig. 5D and E). Meanwhile, the wound healing experiments underlined that when silencing the expression of YWHAG the migration of LUAD cells was significantly alleviated (Fig. 5F and G). In addition, according to the immunofluorescence, with the knockdown of YWHAG, the YP1-positive cells and PI-positive cells exhibited significant increase in both A549 and H1299 cell lines, which unraveled that the high expression of YWHAG in LUAD remarkably inhibited the death of cancer cells (Fig. 5H*–*J). Similarly, there was a measurable increase in the number of apoptotic cells following YWHAG depletion in A549 and H199 cell lines, which was consistent with the above results of immunofluorescence (Fig. 5K*–*N). Furthermore, we validate the cancer-promoting function of YWHAG in xenograft models. As a result, we found that silencing YWHAG decreased the weights and alleviated tumor growth (Fig. 5O*–*Q). Intriguingly, post-YWHAG knockdown decreased the abundance of Ki-67 and YWHAG (Supplemental Digital Content Figure S7E, available at: http://links.lww.com/JS9/E600 and Supplemental Digital Content Figure 5R, available at: http://links.lww.com/JS9/E600). Meanwhile, Supplemental Digital Content Figure 5S, available at: http://links.lww.com/JS9/E600, emphasized a significantly positive correlation between YWHAG and Ki-67. The above results demonstrate that targeting the YWHAG protein may be a reasonable approach to suppress LUAD.

### Inhibition of YWHAG enhances the sensitivity of LUAD cells to pemetrexed and triggers PANoptosis

**Figure 6.**
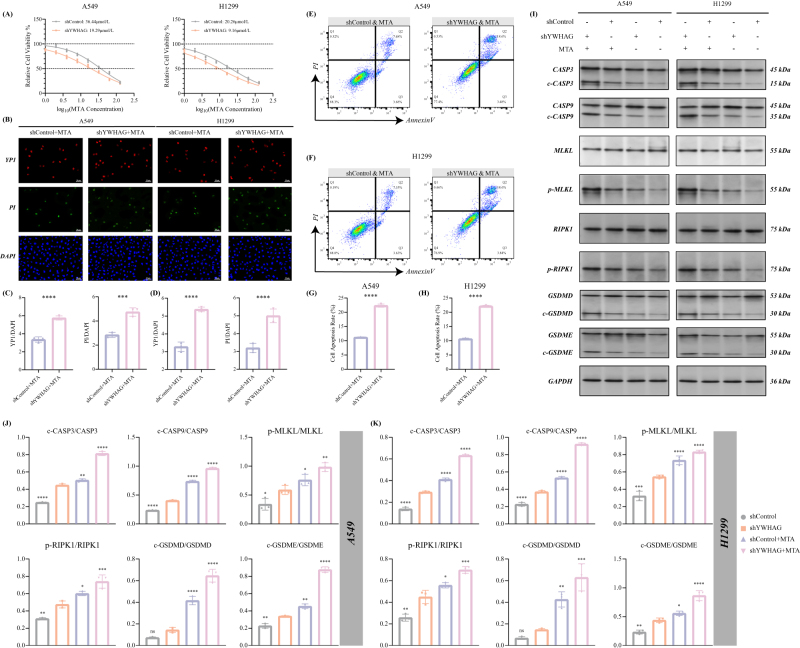
YWHAG decreased the sensitivity to pemetrexed (MTA) and obstructed the occurrence of PANoptosis (*n* = 3/group). (A) The IC50 values of the MTA were calculated across the indicated two cell groups. (B) Representative images of immunofluorescence showed the YP1+ cells (red) and PI+ cells (green) in the labelled cell groups. Relative quantification of the proportion of YP1+ cells and PI+ cells in (C) A549 and (D) H1299 cell lines. (E, F) Representative images for flow cytometric analysis of apoptosis in the labelled cell groups. Relative quantification of the percentage of AnnexinV+PI+ cells in (G) A549 and (H) H1299 cell lines. (I) Immunoblotting of main marker protein expression undergo PANoptosis, including apoptosis, necroptosis, and pyroptosis after YWHAG inhibition in A546 and H1299 cells treated with 1 μM MTA for 48 h. GAPDH was included as a loading control. Subsequent quantifications of these marker proteins’ expression in (J) A549 and (K) H1299 cell lines are exhibited. “-” indicates *P*-value >0.05, *P-value <0.05, **P-value <0.01, ***P-value <0.001, ****P-value <0.0001, by unpaired *t*-test or one-way ANOVA test.

Seeing the crucial role of YWHAG in the development and phenotype of LUAD, we further explore the therapeutic characteristic of YWHAG in LUAD. Pemetrexed (MTA) is one of the most common chemotherapeutics of LUAD, we therefore discussed the influence of YWHAG to MTA therapy. First, with the decrease of the YWHAG expression, the IC50 of MTA similarly declined in the A549 and H1299 cell lines (Fig. 6A and B). Moreover, combination of MTA and YWHAG silence further increased the number of YP1-positive and PI-positive cells compared with using MTA alone (Fig. 6B*–*D). Similar results can be observed via flow cytometry, as evidenced by increased apoptotic cells in the shYWHAG + MTA group (Fig. 6E*–*H). All the above results indicated that the effects of the combination of YWHAG knockdown with MTA treatment significantly increased the number of dead cells.

Considering that YWHAG was one of the elements in our PAN Score prognostic model, we speculated that the YWHAG deficiency in combination with MTA leads to the activation of PANoptosis. Therefore, we further validated the impact of YWHAG silence on PANoptosis during MTA treatment in the molecular level. In terms of apoptosis, we observed that as the expression level of YWHAG decreased and MTA was added, the cleaved forms of apoptosis executioners, CASP3 and CASP9, gradually significantly increased in A549 and H1299 cell lines (Fig. 6I*–*K). Meanwhile, significant increases in phosphorylation of MLKL and RIPK1 were detected, which means the activation of necroptosis (Fig. 6I*–*K). Additionally, as the other member of PANoptosis, pyroptosis, was also found with a growth during the inhibition of YWHAG and MTA treatment, as evidenced by robust cleaved form of GSDMD and GSDME (Fig. 6I*–*K). Overall, we suggested that YWHAG can be regarded as the inhibitor of PANoptosis and may serve as a broad biomarker for susceptibility to MTA-based chemotherapy.

### YWHAG silencing impedes LUAD by reducing vasopressin and prompting PANoptosis

**Figure 7.**
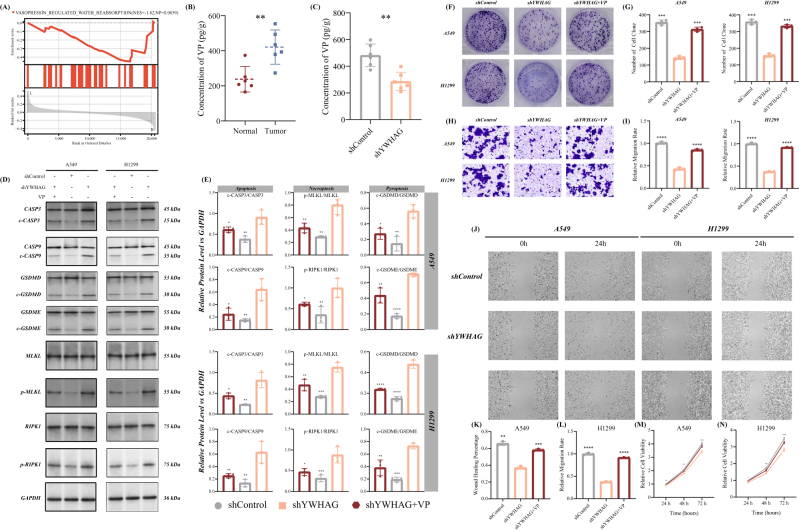
Establishment of the YWHAG-vasopressin (VP)-PANoptosis axis in LUAD. (A) Result of gene set enrichment analysis (GSEA) indicated the most crucial pathways influenced by YWHAG in the TCGA-LUAD cohort. (B) Concentration of VP in the tumor and normal group of our in-house cohort (*n* = 6/group). (C) Concentration of VP before and after the YWHAP knockdown in the xenograft models (*n* = 6/group). (D) Immunoblotting of main marker protein expression undergo PANoptosis, including apoptosis, necroptosis, and pyroptosis after YWHAG inhibition in A546 and H1299 cells treated with 1 μM VP for 48 h. GAPDH was included as a loading control (*n* = 3/group). (E) Subsequent quantifications of these marker proteins’ expression in A549 and H1299 cell lines are exhibited. (F, G) Clonoy formation assays of LUAD in the labelled cell groups after YWHAG inhibition and VP treatment (*n* = 3/group). (H, I) Transwell assays exhibited the invasion phenotype of LUAD in the labelled cell groups after YWHAG inhibition and VP treatment (*n* = 3/group). (J) Representative images of wound healing exhibited the migration phenotype of LUAD in the labelled cell groups after YWHAG inhibition and VP treatment (*n* = 3/group). Relative quantification of the width ratio of the wound in (K) A549 and (L) H1299 cell groups after YWHAG inhibition and VP treatment. Cell viability assays indicated the cell proliferation phenotype of LUAD in (M) A549 and (N) H1299 cell groups after YWHAG inhibition and VP treatment (*n* = 3/group). “-” indicates *P*-value >0.05, *P-value <0.05, **P-value <0.01, ***P-value <0.001, ****P-value <0.0001, by unpaired *t*-test or one-way ANOVA test.

To explore the relationship and potential mechanisms between YWHAG and PANoptosis in LUAD, we first explored the potential downstream pathways regulated by YWHAG. The results of GSEA based on the KEGG database indicated that the VP regulated water reabsorption was significantly enriched in the high-expressed YWHAG group (Fig. 7A). And we detected the concentration of VP between the normal and tumor group in our in-house group, which exhibited a high level in the tumor group (Fig. 7B). Meanwhile, with the inhibition of YWHAG expression, the concentration of VP also decreased (Fig. 7C). And intriguingly, with the inhibition of VP in our mice model, the level of PANoptosis in LUAD were activated, emphasizing the potential relationship between VP and PANoptosis (Supplemental Digital Content Figure S8, available at: http://links.lww.com/JS9/E600). Therefore, we suggested a hypothesis that YWHAG inhibited the activity of PANoptosis in LUAD via increasing the level of VP. Consistent with the hypothesis, YWHAG silencing in A549 and H1299 cells induced the activation or cleaved form of the key protein during the PANoptosis stage, including CASP3, CASP7, MLKL, RIPK1, GSDMD, and GSDME (Fig. 7D and E). However, VP supplementation weakened the activation of PANoptosis by YWHAG knockdown and counteracted the suppressive effects of YWHAG knockdown on these LUAD cell malignant phenotypes (Fig. 7F*–*N). Overall, these findings suggested that the YWHAG-VP-PANoptosis axis played a crucial role in the development and malignant phenotypes of LUAD.

## Discussion

As one of the most common cancer types in recent years, the morbidity of LUAD exhibited a continued growth trend^[15]^. Meanwhile, the 5-year survival rate for advanced LUAD remains below 20%, pointing the poor prognosis of LUAD and the fact that many patients with LUAD do not respond to standard therapies^[16]^. Nowadays, more and more research has claimed the crucial role of PANoptosis in the inhibition of LUAD, which represents a promising avenue to address various challenges in treatment of LUAD, including drug resistance and immune evasion^[17–19]^. However, how to target PANoptosis to improve the prognosis of LUAD patients and enhance their treatment sensitivity remains a challenge. In pursuit of this objective, we exploited diversified prognostic models utilizing 101 algorithm combinations of machine learning with 66 PRGs and the whole-transcriptome profiles of 1411 LUAD biopsy samples from six independent cohorts. Consequently, the prognostic model consisted with 11 genes, PAN Score, derived from the combination of the CoxBoost and RSF, emerged as the superior one, further indicated the crucial prognostic characteristic of PANoptosis in LUAD.

In recent years, several literatures have revealed the prognostic model based on PANoptosis^[20–23]^. However, many of these signatures have been constructed and validated from limited cohorts and the development process is not rigorous or only uses limited algorithms. Our 11 PAN Score genes’ model was developed within the entire transcriptome matrix and encompassed comprehensive algorithm fundamentals. In detail, the PAN Score was derived using a comprehensive machine-learning framework encompassing 101 distinct algorithm combinations, significantly enhancing model robustness and reducing algorithm-dependent bias compared to studies employing single or few methods. And the PAN Score demonstrated superior and consistent prognostic performance across six independent cohorts, outperforming 47 previously published mRNA signatures, highlighting its unprecedented generalizability. Moreover, we provide a deep mechanistic link between PAN Score and an immunosuppressive TIME, extensively validated using six distinct immune deconvolution algorithms and histopathological assessment, a level of immune context integration often lacking in prior PANoptosis signatures. CYCS, as one of the initiators of apoptosis, functions as a central component of the electron transport chain in mitochondria^[24]^. Ying *et al*^[25]^ pointed that the up-regulation of CYCS can prompt the apoptosis in tumor cells to control the progress of GBM. And we also confirmed that the low expression of CYCS was a risk factor of LUAD. CASP10, FADD, and TNFRSF1A1 were the direct participants of apoptosis and promote the form of the death-inducing signaling complex, which had become the widely recognized anti-tumor molecules^[26–28]^. Moreover, DFFB, the active component of DNA fragmentation factor (DFF), has been found to trigger both DNA fragmentation and chromatin condensation during apoptosis^[29]^. In the research of Kulbay *et al*^[30]^, DFFB was observed with down-expressed in the cell lines of breast cancer, ovarian cancer, lung cancer, and glioblastomas, whose absence exhibited the chemoresistance to antimetabolites. Additionally, abundant research has pinpointed that YWHAG can be regarded as a carcinogenic factor associated with apoptosis^[31,32]^. IL1A, a cytokine produced by monocytes, control many different cellular functions including proliferation, differentiation and cell survival/apoptosis but are also involved in several pathophysiological processes^[33]^. And IL1A also involves in the regulation of immunotherapy and TME of LUAD^[34, 35]^. MLKL and RIPK3 were two crucial mediators of necroptosis induced by tumor necrosis factor (TNF), playing a wide and key role in the inhibition of cancers^[36]^. TFDP1 can combine with E2F to form E2F1-DP complex to mediate both cell proliferation and apoptosis, which is associated the occurrence and development of hepatocellular carcinoma and colorectal cancer^[37, 38]^. And TICAM1 has been confirmed that can strengthen the efficiency of immunotherapy in cancer via combining with TLR3^[39]^. Overall, these findings demonstrated that our PAN Score, established by the 11 genes, reflects the influence of PANoptosis associated with LUAD progression, which could explain its increased predictive precision over clinicopathological markers and existing LUAD signatures.

Apart from emphasizing a prognostic model, PAN Score, for stratifying LUAD patients and implementing the personalized therapy strategies, we also ensured YWHAG as a crucial therapeutic target for these patients. YWHAG, as a member of the 14-3-3 protein family and among the most abundant proteins in cells, is highly conserved and participates in the regulation of tumor cells’ growth, cell cycle, apoptosis, migration, and invasion^[31]^. More and more evidence has revealed that YWHAG plays a carcinogenic role in several cancer types^[40–43]^. For example, Lee *et al*^[31]^ pinpointed that the deficiency of YWHAG can prevent the development and metastasis of cancers via disrupting the EMT-associated network and inducing oxidative cell death. In addition, the deficiency of YWHAG can suppress the surface expression of ANO1 to inhibit the progress of glioblastoma cells^[42]^. Although slight research indicated that YWHAG can inhibit the development of LUAD, the influence and regulation of YWHAG in the progress and therapy of LUAD remain elusive. In our research, we observed the high expression of YWHAG mRNA and protein in LUAD biopsy samples of public and our in-house cohorts, which was associated with the poor prognosis of LUAD patients. Meanwhile, we found that YWHAG knockdown cell lines led to reduce the malignant phenotypes of LUAD, consistent with the finding of Wang *et al*^[44]^ and Fu *et al*^[45]^. Interestingly, our analysis first indicated that the deficiency of YWHAG can enhance the sensitivity to a common chemotherapeutics of LUAD, MTA, further emphasizing the crucial role of YWHAG in the development and treatment of LUAD. Overall, our data suggest that YWHAG is a promising prognostic indicator, and YWHAG inhibition may represent a potential treatment strategy for chemotherapy resistance in LUAD treatment. However, further investigation is needed to elucidate the underlying molecular mechanism of the oncogenic role of YWHAG in LUAD.

Diversified gene clusters associated with PANoptosis exhibited significant correlation with the TILs, expression of ICKs, and response to immunotherapy or chemotherapy of cancers^[46, 47]^. The interplay between PANoptosis and the immune microenvironment represents a pivotal axis in LUAD progression and therapy resistance. Our findings demonstrate that patients with high PAN Scores exhibit a “cold” TIME characterized by reduced immune cell infiltration and suppressed activity of key ICK regulators. The elements in the PAN Score, such as IL1A and TNFRSF1A, are known regulators of inflammatory signaling. Their dysregulation in high PAN Score tumors may impair cytokine secretion (e.g. IL-2, IFN-γ) critical for T-cell activation and recruitment, thereby creating an immunosuppressive niche. Meanwhile, CYCS and CASP10, integral to mitochondrial apoptosis, may influence immunogenic cell death (ICD) and subsequent antigen cross-presentation by dendritic cells. High PAN Score tumors might evade immune detection by suppressing ICD-related damage-associated molecular patterns. The negative correlation between PAN Score and PD-L1/CTLA-4 expression suggests that PANoptosis-related pathways may epigenetically silence ICK genes, though this requires experimental validation. These mechanistic insights align with recent studies linking PANoptosis to NLRP3 inflammasome activation and T-cell exhaustion, but our multi-omics approach uniquely identifies LUAD-specific vulnerabilities. Future work should explore whether PAN Score-associated immune suppression is reversible through targeted therapies (e.g. IL-1A blockade or necroptosis inducers).

Targeting the PANoptosis-related pathway brings hope for cancer treatment. For example, ADAR1 plays a crucial role in prompting the progress of tumors by control the immune response and PANoptosis induced by ZBP1^[9]^. And another study indicated that the crosstalk between PPM1B and USP10 can inhibit YBX1-mediated PANoptosis to promote the chemoresistance of oxaliplatin in gastric cancer^[48]^. However, as a member of PAN Score in LUAD, the specific role and potential mechanism of YWHAG in regulating PANoptosis remain unclear. Our research confirmed that the knockdown of YWHAG can activate apoptosis in LUAD. And YWHAG can be regarded as an inhibitor of PANoptosis in LUAD through our analysis. Subsequently, via the functional enrichment analysis, VP was identified as the key mediator between YWHAG and PANoptosis in LUAD. VP, traditionally recognized for its antidiuretic function, has increasingly been implicated in tumorigenesis, including lung cancer^[49]^. Specifically, VP can act as a mitogen when binding to V1a and V2 receptors, stimulating the proliferation of cancer cells^[50]^. Moreover, VP has been shown to enhance the expression of angiogenic factors, thus promoting tumor vascularization and subsequent tumor growth^[49]^. In our research, the knockdown of YWHAG can decrease the concentration of VP in LUAD cells to strengthen PANoptosis and inhibit the progress of LUAD. However, there are still aspects that require greater focus regarding the role of YWHAG-VP-PANoptosis axis in other types of cancers, as well as its impact on various chemotherapy and immunotherapy treatments, particularly those that are resistant to currently available therapies.

Nevertheless, the limitations of our study are worthy to be mentioned. As a retrospective study, there were some biases in our analysis, emerging the need of follow-up prospective trial for further verification of the predictive accuracy of our PAN Score. And the reliance on retrospective public cohorts (e.g. TCGA) introduces potential biases, such as uneven representation of early-stage vs. advanced LUAD and under-sampling of ethnic/geographic diversity. Second, the machine learning framework, while robust, risks overfitting due to the high dimensionality of transcriptomic data. External validation in prospectively collected, therapy-naïve cohorts is essential before clinical translation. Third, except for YWHAG, the biological functions of other genes in the PAN Score have not yet been investigated. Fourth, the *in vitro* and xenograft models used here lack human immune components, limiting their ability to recapitulate TIME dynamics. Co-culture systems with patient-derived immune cells or humanized mouse models would better assess therapeutic potential. Despite these limitations, our study establishes a foundational framework for targeting PANoptosis in LUAD. We strongly advocate for prospective trials stratifying patients by PAN Score to validate its clinical utility and mechanistic hypotheses.

## Conclusion

In summary, our research demonstrated that the exploiting of PAN Score brought gospels to the accurate prediction of prognosis and the development of personalized therapies for LUAD patients. Meanwhile, we also revealed that YWHAG protein is a potential therapy target for these patients. The deficiency of YWHAG controls the secretion of VP to activate the PANoptosis, finally inhibiting the development of LUAD. These findings highlight the significance of YWHAG-VP-PANoptosis axis as a prognostic indicator and a promising therapeutic target for LUAD. Targeting the above axis offers a promising therapeutic avenue, representing a potential supplementary treatment for high-risk LUAD patients.

## Data Availability

Public data used in this work can be acquired from the TCGA (https://portal.gdc.cancer.gov/) and GEO (http://www.ncbi.nlm.nih.gov/geo/). The raw experimental data and analysis codes supporting the conclusions of this article will be made available by the corresponding author.

## Research registration unique identifying number (UIN)

This study has been registered in the Research Registry with the UIN researchregistry10860 (https://www.researchregistry.com/).

## Assistance with the study

We sincerely acknowledge the contributions from the public platform BioRender (https://www.biorender.com/) for the contribution of Graphical Abstract.
